# Dog Theft: A Case for Tougher Sentencing Legislation

**DOI:** 10.3390/ani8050078

**Published:** 2018-05-22

**Authors:** Lauren K. Harris

**Affiliations:** Dogs Trust, 17 Wakley St, London EC1V 7RQ, UK; Lauren.Harris@dogstrust.org.uk

**Keywords:** law, companion animals, dogs, human-animal bond, ethics

## Abstract

**Simple Summary:**

The Sentencing Council (England and Wales) currently considers dogs to be “property”. This means that if someone steals a dog, they may be punished in the same way as someone who steals a non-living object, like a mobile phone or a piece of furniture. This review argues that losing a dog is very different to losing a non-living object, and that many people consider their dog to be a friend or a family member, not just a “possession”. The review concludes that that people who steal dogs should be punished in a way that reflects the emotional harm that can be caused to victims of dog theft.

**Abstract:**

Dogs, and other companion animals, are currently classed as “property” in theft sentencing legislation for England and Wales. This means that offenders who steal dogs are given similar sentences to those that steal inanimate objects. This review presents the argument that the penalty for dog theft should be more severe than for the theft of non-living property. Evidence of the unique bond between dogs and humans, and discussion of the implications of labelling a living being as mere “property” are used to support this argument. The review concludes that the Sentencing Council’s guidelines should be amended so that offences involving the theft of a companion animal are deemed to be a Category 2 offence or above. The review further proposes that “theft of a companion animal” should be listed in the Sentencing Council’s guidelines as an aggravating factor.

## 1. Introduction

At the time of writing, dogs are classed as “property” in Sentencing Council guidelines for theft offences for England and Wales. This means that the sentences given to offenders who commit dog theft are often similar in severity to those handed down for the theft of an inanimate object, such as a mobile phone or a piece of furniture. In a recent consultation on the theft sentencing guidelines [1], The Stolen and Missing Pets Alliance suggested additional guidelines to cover the theft of companion animals. The Sentencing Council ruled against this proposal, stating that: “The harm caused by the theft of a much-loved pet can be taken into account as part the assessment of harm, in which the factor of “emotional distress” would enable the court to reflect any significant additional harm caused by the theft”. However, the penalty for dog theft is often decided based on the monetary value of the dog, and not the emotional value to the dog’s guardian. The current theft offences guidelines published by the Sentencing Council [2] classify the level of harm caused by theft into four categories. For the theft to be classed as Category 1 or 2 (the most severe categories), the “property” stolen must have a monetary value over £500, regardless of how much non-financial harm (including emotional harm) is caused. Although some dogs have a financial value which far exceeds £500, many dogs have little or no monetary value. The theft of a dog valued at less than £500 can only be classed as a Category 3 or 4, which means less severe sentences are recommended in the guidelines. The most severe sentence recommended for stealing a dog that is worth less than £500 is two years custody.

This review presents evidence regarding the special bond between humans and dogs, which places them above mere “property”, and argues that there are grounds for amending the guidelines to encourage tougher sentences for dog theft. Although dogs are the primary focus of the review, reference is often made to “companion animals”, this is because it seems arbitrary to argue for more severe sentences for the theft of dogs and not other companion animals such as cats.

## 2. The Human-Canine Bond

“Attachment theory” is an explanation of how humans form close emotional relationships with one another. The theory was originally developed by psychologists John Bowlby and Mary Ainsworth in order to describe the bond between mother and child [3]. Attachment theory has been adapted and expanded in order to describe attachment to a romantic partner [4], and attachment to friends and peers [5,6]. More recently, it has also been applied to the bond between dogs and their guardians [7,8].

Similar measures of attachment security can be applied to dog-human relationships as to human-human relationships. How secure someone feels in a relationship can be determined by exploring whether they worry about being rejected, need constant reassurance of their partner’s love, or feel they can confide in their partner. Using these measures, it has been found that in some cases relationships with companion animals are “more secure” than attachments to romantic partners [9]. The reported security of guardians’ relationships with their companion animals may be explained by the perception that a non-human animal can provide unconditional love, and does not judge or criticise [10]. There is evidence that human-human relationships are becoming less secure and more transient in modern society, and some authors have linked reduced security in human relationships to the increase in companion animal guardianship and increased reliance on the companionship of non-human animals [11].

Companion animal guardians often report that they consider their companions to be members of the family. For example, one survey found that 87%, and another found that 99%, of respondents considered their companion animals to be family members (Cain 1983 and Voith 1985; cited in Reference [12]). Another survey found that 95% of companion animal guardians regarded their animals as “friends” (Stallones et al., 1988; cited in Reference [12]). The dog-human bond has been likened to that found between a parent and child; one study used functional magnetic resonance imaging (FMRI) to scan mothers’ brains while they viewed a series of photographs of either their child or of their dog. This study found that the brain regions activated when mothers viewed images of their children were similar to those activated when they viewed pictures of their dog. These brain regions were those associated with “emotion, reward, affiliation, visual processing and social cognition” [13].

The dog-human bond may be explained by the long history of canine domestication; it is estimated that dogs diverged from their wild ancestors over 100,000 years ago [14]. During this time, dogs have adapted physically and behaviorally for living with humans, and a unique social bond has developed between the species. For example, dogs have demonstrated that they are “more skillful than great apes” when it comes to performing tasks that rely on communicating with humans, particularly those that involve interpreting visual cues, such as pointing, given by humans to indicate the position of hidden rewards [15,16].

## 3. How Do Dogs Benefit Humans?

At the beginning of the domestication process, dogs would have predominantly served functional roles, such as herding and hunting [17], but in modern times, many dogs are kept solely for companionship. This has led some to question the benefit of dog companionship for people, with some authors even describing the relationship between dogs and people as “parasitic” [18].

However, even aside from instances where dogs have an obvious benefit to their guardians (e.g., service dogs, medical assistance dogs, or therapy dogs), there is evidence that dogs can have a positive impact on their guardians’ lives. Studies suggest that having a dog can improve health through encouraging exercise [19,20,21]. Dogs (and companion animals in general) may also improve psychological wellbeing. For example, there is evidence that having a dog can reduce risk of depression; a recent survey of people living with HIV found that non-dog guardians had 3 times higher odds of depression compared to current dog guardians [22]. There is also evidence that companion dogs can reduce anxiety in children with autism spectrum disorder [23], and interaction with a dog has been found to reduce anxiety and increase wellbeing and positive mood in University students [24].

Loneliness has been described as an “epidemic” in modern day society [25], and can have a significant impact on public health, with research suggesting that social isolation is a risk factor for premature mortality [26]. Knight and Edwards reported that owning a dog can reduce loneliness in elderly populations by promoting a social support network between dog guardians [27]. Other research has shown that women living entirely alone are significantly more likely to report being lonely than those living with a companion animal, suggesting that non-human animals are able to compensate for the absence of human companionship [28].

It has been acknowledged by some that the positive effects of companion animals on mental health are too inconsistent to be conclusive [29]. However, others have countered that these inconsistencies are due to the fact that there are many other variables involved in the manifestation of mental ill health, and it is likely that certain demographics, e.g., women and single adults, are more likely benefit from dog guardianship than other groups [30].

## 4. How Can the Theft of a Dog Impact Their Guardian?

The evidence above demonstrates the potential strength of the bond between guardian and dog, and when a strong relationship bond is broken it is likely to cause emotional trauma. Studies have shown that the grief experienced by guardians following the loss of a companion animal parallels that experienced following human bereavement [12,31]. In the field of psychology, specifically the study of grief and bereavement, there is a distinct term for the grief felt in response to a missing person: “ambiguous loss”. Ambiguous loss is often experienced by the friends and families of missing persons and is characterized by a lack of closure or understanding [32]. The lack of finality associated with ambiguous loss, and the added pain of “not knowing”, means that the grief related to missing persons can be longer lasting, and in some cases even more traumatic than bereavement following death [33,34]. It is therefore likely that the emotional impact of a dog going missing is at least as significant as the emotional impact of companion animal bereavement.

Another factor that may arise following the loss of an animal companion is “disenfranchised grief” [35]. Disenfranchised grief is a term used to describe grief which is not acknowledged by society, this often results in a situation where the griever feels that they cannot grieve openly or speak about their feelings for fear that they will not be taken seriously [36]. People mourning the loss of a companion animal will often be faced with unsympathetic comments like “it’s only a dog” and be expected to just “get over it” [35]. This lack of empathy may dissuade those suffering from the loss of a dog from talking about their grief, which could give an unrealistic view of how deeply the loss has affected them. Reluctance of those grieving the loss of a companion animal to talk about their grief could perpetuate the idea that losing a companion animal is trivial compared to other types of loss. Finally, other’s refusal or inability to acknowledge someone’s grief may extend the time taken for the griever to recover, and make it more difficult to come to terms with their loss [37].

The loss of a dog can be particularly hard on those who have little social support in the form of human family or friends. In interviews with elderly people who have dogs, many described their dogs as “surrogate” family members, who helped them cope in difficult emotional times such illness and widowhood [27].

At the time of writing, only one study that directly measured the emotional impact of dog theft on guardians could be identified. This study involved in depth interviews with dog guardians who had experienced dog theft, and revealed a wide range of psychological and social impacts on the victims. For example, approximately 30% of interviewees reported feelings of “loss, grief or mourning”, approximately 48% described themselves as “devastated”, and approximately 37% suffered from “severe psychological or physiological effects” after their dog was stolen. The interviews also found that a high percentage of victims (78%) reported that their social life had been negatively affected, while around 41% described negative effects on their family or work life, with one participant reporting that they had ended up losing their job due to taking too much time off to search for their dog [38].

It is difficult to estimate the financial impact of dog theft, clearly it depends largely on factors such as the pedigree of the dog, their ability to compete in shows, their skills as a working dog, and their guardian’s intentions to breed from them. However, aside from the actual value of the dog, theft may incur additional costs to their guardian and wider societal costs through efforts to locate them. These costs may include printing posters, time taken off work, travel costs and providing a monetary reward for the dog’s safe return. It has been estimated that the total cost of the search for a missing dog can exceed £4000 [39]. While is it true that many guardians may not be willing or able to spend this much on locating their dog, there are documented cases where guardians have far exceeded this estimate: For example, one guardian re-mortgaged their house to offer a £10,000 reward, while another gave up their business to dedicate themselves full time to searching for their dog [39].

## 5. Problems Associated with Treating Companion Animals as “Property”

Animal law scholars and advocates have highlighted the impact of the language we use to refer to non-human animals on the way humans treat members of other species. By equating animals to “things” or “property” to be owned, we are denying them the right to be considered sentient beings, distinct from inanimate objects [40,41,42]. If the law states that animals are worth no more than property, that sentiment may filter down to people who will use it as justification to mistreat animals, and people who commit crimes against animals will not be punished appropriately [43].

Theft is not the only area where British law considers dogs mere “property”. If someone kills or injures someone else’s dog, the perpetrator may be charged under the Criminal Damage Act 1971; the same sort of charge they would face if they broke someone’s TV, or smashed a car windshield [44]. The reason that some cases where an animal is injured may be charged under the Criminal Damage Act 1971 rather than the Animal Welfare Act 2006 (for animal cruelty) is that criminal damage is often treated as a more serious crime; for example, animal cruelty cases can only be heard and sentenced in a Magistrates Court, but criminal damage cases can reach the Crown Court [45]. This issue was brought to public attention in 2016, when a police dog named Finn was stabbed in the head and chest while on duty. Finn’s story made headlines, and the fact that the perpetrator was only charged with “damaging police property” for injuring Finn sparked a campaign known as “Finn’s Law”. A petition calling for police dogs (and horses) to be given the same legal protection as human police officers reached over 100,000 signatures, which meant it could be discussed in parliament [46]. The debate took place in November 2016, the result was that the animal cruelty sentencing guidelines were revised so that “animal being used in public service” was added as an aggravating factor. The animal cruelty sentencing guidelines recommend a maximum sentence of 26 weeks custody [47], however, judges may award greater sentences if they feel the offence is serious enough, particularly if there are aggravating factors involved.

## 6. Legal Cases Where the Categorization of Dogs as “Property” Has Been Questioned in Court

There have been a number of instances where courts have found the classification of dogs as property inadequate. Some examples are listed below, with excerpts from the case notes. These cases were carried out in the U.S. and are therefore unlikely to have any legal significance to UK law. However, since U.S. law also classes companion animals as “property” the comments below suggest that there is some dissatisfaction with this classification among legal professionals. In some cases, the courts awarded damages for loss of companionship, but in others the courts were unable to award sufficient damages due to the laws that classify dogs as property, despite their beliefs that dogs should be treated differently.

### 6.1. Myers v. City of Hartford. 853 A.2d, 625 (Conn. App. Ct. 2004)

“Labelling a pet as property fails to describe the emotional value human beings place on the companionship that they enjoy with such an animal. Although dogs are considered property… this term inadequately and inaccurately describes the relationship between an individual and his or her pet.”

### 6.2. Rabideau v. City of Racine. 627 N.W.2d 795 (Wis. 2001)

“At the outset, we note that we are uncomfortable with the law’s cold characterization of a dog, such as Dakota, as mere “property.” Labelling a dog “property” fails to describe the value human beings place upon the companionship that they enjoy with a dog. A companion dog is not a fungible item, equivalent to other items of personal property. A companion dog is not a living room sofa or dining room furniture. This term inadequately and inaccurately describes the relationship between a human and a dog.”

### 6.3. Carl Bueckner v. Anthony (Tony) Hamel and Kathy Collins 886 S.W.2d 368 (Tex. App. 1994)

“Society has long since moved beyond the untenable Cartesian view that animals are unfeeling automatons and, hence, mere property. The law should reflect society’s recognition that animals are sentient and emotive beings that are capable of providing companionship to the humans with whom they live. In doing so, courts should not hesitate to acknowledge that a great number of people in this country today treat their pets as family members. Indeed, for many people, pets are the only family members they have. Losing a beloved pet is not the same as losing an inanimate object, however cherished it may be. Even an heirloom of great sentimental value, if lost, does not constitute a loss comparable to that of a living being. This distinction applies even though the deceased living being is a nonhuman.”

### 6.4. Corso v. Crawford Cat and Dog Hospital Inc 415 N.Y.S.2d (182 N.Y.City Civ.Ct., 1979)

“This court now overrules prior precedent and holds that a pet is not just a thing but occupies a special place somewhere in between a person and a piece of personal property.” “In ruling that a pet such as a dog is not just a thing I believe the plaintiff is entitled to damages beyond the market value of the dog. A pet is not an inanimate thing that just receives affection it also returns it” “This decision is not to be construed to include an award for the loss of a family heirloom which would also cause great mental anguish. An heirloom while it might be the source of good feelings is merely an inanimate object and is not capable of returning love and affection. It does not respond to human stimulation; it has no brain capable of displaying emotion which in turn causes a human response. Losing the right to memorialize a pet rock, or a pet tree or losing a family picture album is not actionable. But a dog that is something else. To say it is a piece of personal property and no more is a repudiation of our humaneness. This I cannot accept.”

## 7. Previous Proposals for a New Legal Status for Non-Human Animals

Many legal and philosophical scholars have called for new legal statuses for animals which distinguish them from inanimate property. It should be noted that the authors cited in this section are predominately from the U.S. As such, some of the references to specific legislation in these texts are relevant only to the U.S. However, since the primary arguments of these articles are rejecting the status of animals as property, often from a philosophical perspective, they are still relevant to this review. Some authors completely reject the idea that animals should be treated as “property” by the law, arguing that this classification ignores the interests of animal themselves, and only deals with the animal’s value to humans [48]. Some of those who reject the property status of animals argue that at least some other non-human species should be granted “legal personhood” [49,50]. Authors who take this view often reject the idea that any animal be treated as property, not just companion animals.

Other authors argue that companion animals need not completely lose their status as property, but instead be classified as “an enhanced type of property—one that recognises their important differences from inanimate property” [51]. Favre [52] posed the idea that animals should be classed as “living property”, and that the legal duty towards animals should be viewed similarly to the duty of parents to care for a child. Some have argued that as well as legislation being changed to classify animals as “semi-property”, claimants should be able to claim for “loss of companionship” on top of any compensation for loss of monetary value [53]. White [54] argued that language change in legal guidelines (for example, changing the term animal “owner” to “guardian”) could have an important impact on the way companion animals are treated in society: “Symbolic language change will educate the public and encourage people to think of and treat their pets more like family and household members and less like disposable property”.

Divorce courts in Australia have recognized that the current inclusion of companion animals in the chattel or property category does not reflect modern ideals. This has led some to call for new legislation dictating how to deal with companion animals in divorce settlements; recent proposals for this legislation are based largely on child custody laws [55].

Despite the number of legal experts and scholars that have called for changes to the legal status of animals, there have been few examples where legislation has actually been changed. One of these examples is the 2015 amendment to French civil law which meant that companion animals are now considered to be “living beings gifted sentience,” rather than “movable property” [56]. Another example is the 2016 amendment to Alaska’s divorce statutes, which means that companion animals are treated more like children than inanimate property in custody disputes. This amendment specifically requires the animal’s wellbeing to be taken into account when making custody decisions and will allow joint custody agreements to be made [57].

## 8. Conclusions

In conclusion to this review, based on the evidence presented, it is proposed that the Sentencing Council should reconsider amending existing guidelines to ensure that all cases of companion animal theft are treated equally seriously, regardless of monetary value. This proposal can be achieved in two ways.

### 8.1. Category Grading

The harm section of the guidelines is amended so that any offence involving the theft of a companion animal is deemed to be a Category 2 offence or above. A starting point of two years custody for a culpability Category A offence, one year for Category B and a High Level Community Order for a Category C offence are considered a more appropriate sentences for any person convicted of stealing a companion animal (See Appendix A).

### 8.2. Other Aggravating Factors

“Theft of a companion animal” is added to the list of other aggravating factors. This adaptation would increase the seriousness of the offence, allowing the courts to impose tougher punishments that properly reflect the anguish that these offences cause to victims.

There seems to be a degree of incongruence between the law and society in the way that companion animals are viewed. As a society, we generally recognize that a relationship with a companion animal is entirely different to any attachment one might feel towards an inanimate object. The law, on the other hand, classifies non-human animals in the same category as personal property, which trivializes the emotional harm that can be caused by the theft of a companion animal.

Although this review has focused largely on dogs, the author believes there is not sufficient evidence to justify elevating the legal status dogs above other companion animal species, for example cats. It is impossible to predict whether a dog guardian would be more or less distressed by the loss of their companion compared to a cat guardian. Therefore, the recommendations laid out above apply to all companion animal species and are not exclusive to dogs.

## Appendix A

The sentencing council determines the level of culpability by “weighing up all the factors of the case to determine the offender’s role and the extent to which the offending was planned and the sophistication with which it was carried out”. Culpability can be categorized as “high culpability” (Category A), “medium culpability” (Category B), “lesser culpability” (Category C) [2].
